# Recent Advances in Copper Catalyzed Alcohol Oxidation in Homogeneous Medium

**DOI:** 10.3390/molecules25030748

**Published:** 2020-02-09

**Authors:** Telma F. S. Silva, Luísa M. D. R. S. Martins

**Affiliations:** Centro de Química Estrutural, Instituto Superior Técnico, Universidade de Lisboa, Av. Rovisco Pais, 1049-001 Lisboa, Portugal

**Keywords:** copper, complexes, homogeneous catalysis, peroxidative, aerobic, oxidation of alcohols

## Abstract

The development of sustainable processes and products through innovative catalytic materials and procedures that allow a better use of resources is undoubtedly one of the most significant issues facing researchers nowadays. Environmental and economically advanced catalytic processes for selective oxidation of alcohols are currently focused on designing new catalysts able to activate green oxidants (dioxygen or peroxides) and applying unconventional conditions of sustainable significance, like the use of microwave irradiation as an alternative energy source. This short review aims to provide an overview of the recently (2015–2020) discovered homogeneous aerobic and peroxidative oxidations of primary and secondary alcohols catalyzed by copper complexes, highlighting new catalysts with potential application in sustainable organic synthesis, with significance in academia and industry.

## 1. Introduction

In organic synthesis, the oxidation of primary and secondary alcohols to the corresponding carbonyl compounds, aldehydes (or carboxylic acids) and ketones, respectively, plays a central role due to the wide use of these commodities as precursors and intermediates for fine chemistry (mainly drugs, vitamins and fragrances) [1,2,3].

Classic oxidation methods use stoichiometric quantities of inorganic oxidants, such as manganese dioxide, chromium(VI) or the Swern or Dess—Martin reagents [4], which are often toxic and generate considerable amounts of by-products. Moreover, many of the early found catalytic systems suffer from high reagent cost, instability, employment of hazardous metals or oxidants, harsh reaction conditions, operational complexity, functional group incompatibility or production of non-processable wastes.

**Scheme 1:**
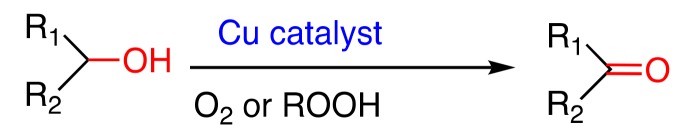
Selective oxidation of primary (R_1_ = H, R_2_ ≠ H) and secondary (R_1_, R_2_ ≠ H) alcohols.

Fortunately, the state-of-the-art in alcohol oxidation research is nowadays by far better. Many catalytic methods that can be used to oxidize alcohols by using peroxides or dioxygen as alternative oxidants are now known, and the implementation of a transition metal-based catalyst in combination with them represents emerging sustainable alternatives to the traditional procedures [1,5] (Scheme 1). Typically, selective peroxidative oxidations of alcohols involve early transition elements in high-oxidation states and peroxometal complexes as the active oxidants. Aerobic oxidations, in contrast, involve oxidative dehydrogenation that is generally catalyzed by late-transition elements.

The development of efficient oxidation systems by using economic and less hazardous catalysts, oxidants and solvents, under mild conditions, has become a relevant aim for catalytic-processes designing, and Cu-containing compounds are of a high potential interest among transition metal catalysts. In fact, copper is cheap and widespread in nature (attractive from the industrial viewpoint) and is present in the active sites of many metalloproteins, particularly in enzymes [6,7] (e.g., particulate methane monooxygenase, galactose oxidase, hemocyanin, cytochrome c, catechol oxidase, laccases or superoxide dismutase). Mononuclear copper complexes, in combination with 2,2,6,6–tetramethylpiperidine-1-oxyl radical (TEMPO), have emerged as one of the most effective catalysts for selective alcohol oxidation, and these catalytic systems are frequently considered the biomimetic functional model of the galactose oxidase enzyme [8]. The efficiency of the latter has led to extensive research to mimic the active sites of this enzyme, leading to a variety of Cu/TEMPO catalytic systems being reported over the years. Thus, copper has drawn particular attention in catalyst design, and exciting research in the realm of coordination chemistry has been reported [8,9].

Nevertheless, numerous challenges remain in the progress of copper-catalyzed aerobic or peroxidative alcohol oxidations with high catalytic activity and selectivity as alternatives for conventional stoichiometric oxidation methods. Since a myriad of procedures are available for the oxidation of alcohols, practicality plays an important role in any new method. This includes mild reaction conditions, low pressures of O_2_ (especially in flammable organic solvents) or low amounts of peroxide, low catalyst loading and low temperatures (preferably room temperature), and the avoidance of costly/toxic additives (co-catalyst, promotor, etc.). Other key challenges are functional group tolerance and the ability to chemoselectively oxidize the alcohol in the presence of other groups susceptible to oxidation. 

In this minireview, the most recent advances (2015–2020 period) in the peroxidative or aerobic oxidation of primary and secondary alcohols, using copper catalysts in homogeneous medium, are addressed.

## 2. Peroxidative Oxidation of Alcohols

### 2.1. Oxidation with Hydrogen Peroxide

Peroxidative oxidations of alcohols are typical model reactions due to their importance and generality; inexpensive, hydrogen peroxide or *tert*-butyl hydroperoxide oxidants, and simple procedures are usually involved. As peroxides can be commercially obtained in aqueous solutions, attempts to improve the sustainability of alcohol oxidations address the use of water as solvent. Therefore, recently, new water-soluble copper complexes have been synthesized, and their ability to act as catalysts for the oxidation of alcohols in aqueous medium have been evaluated. Hydrogen peroxide, the most environmentally friend peroxide, was always the selected oxidant. Indeed, in the environment, hydrogen peroxide, which does not evaporate from water or adsorb to soil, is quickly decomposed by biological and/or chemical processes, to form water and oxygen.

**Figure 1:**
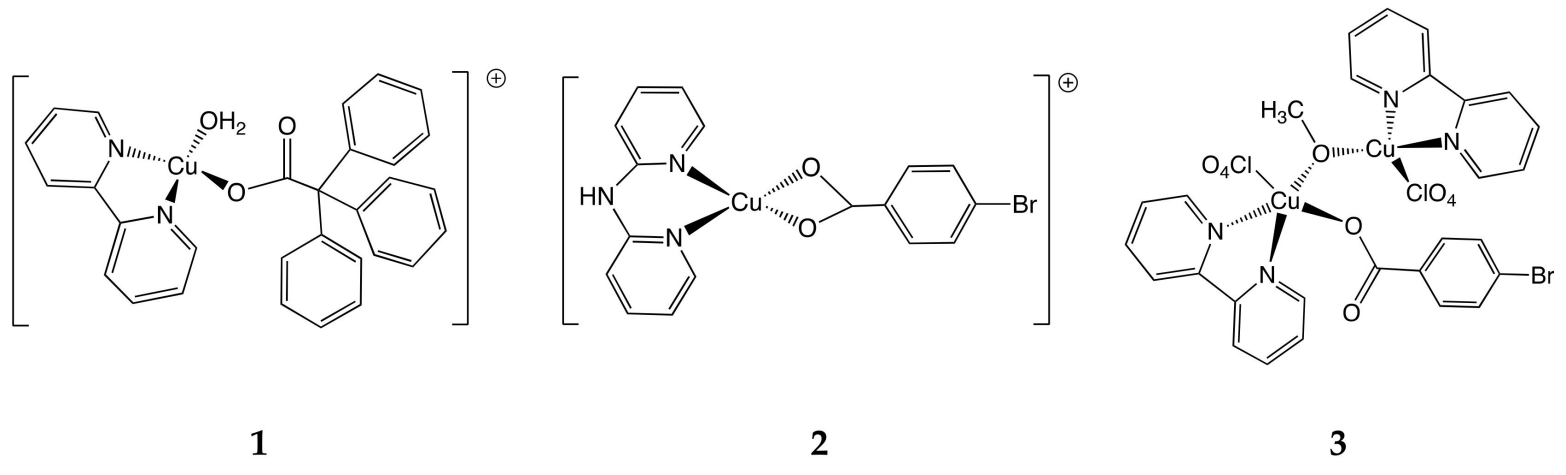
Mononuclear Cu(II) complexes [Cu(OOCC(C_6_H_5_)_3_)(bipy)(H_2_O)][ClO_4_](CH_3_OH) (**1**), bipy = 2-2′-bipyridyl) and [Cu(OOC(C_6_H_5_)Br)(C_10_H_9_N_3_)][ClO_4_] (**2**), and the di-nuclear Cu(II) complex [Cu_2_(OOCC_6_H_4_Br)(OCH_3_)(bipy)_2_(ClO_4_)_2_] (**3**).

In 2017, Kani and coworkers reported the use of the water-soluble mononuclear Cu(II) complex [Cu(OOCC(C_6_H_5_)_3_)(bipy)(H_2_O)][ClO_4_](CH_3_OH) (**1**, bipy = 2-2′-bipyridyl, Figure 1) as catalyst precursor for the oxidation of primary (benzyl alcohol or 1-heptanol) and secondary (1-phenylethanol, 3-pentanol or 2-octanol) alcohols with H_2_O_2_ in water (Table 1). Complex **1** exhibited high catalytic activity toward the oxidation of benzyl alcohol to benzaldehyde as the only product (97% yield after 6 h, entry 1, Table 1) [10] in low load (1 mol% vs. substrate) and at a moderate temperature (70 °C), whereas a more modest activity toward the production of acetophenone or 3-pentanone was observed for the same catalyst (e.g., 73% after 6 h and 100% yield of acetophenone after 24 h; entries 4 and 5, Table 1). Later, the same researchers investigated [11] the catalytic activity of the new water-soluble Cu(II) complex bearing 4-bromobenzoate/2,2′-dipyridylamine [Cu(OOC(C_6_H_5_)Br)(C_10_H_9_N_3_)][ClO_4_] (**2**, Figure 1) for the oxidation of the above alcohols, using the same reaction conditions. Moreover, **2** also exhibited high selectivity toward benzaldehyde, but only 71% of benzyl alcohol conversion after 6 h (entry 10, Table 1) was achieved. The observed behavior of **1** toward the oxidation of secondary alcohols was also found for **2**. For example, complete conversion of 1–phenylethanol to acetophenone was reached only after 24 h (86% after 6 h; entries 14 and 13, respectively, Table 1).

In 2018, Ünver et al. reported the homogenous oxidation of primary and secondary alcohols catalyzed by the related water-soluble di-nuclear Cu(II) complex [Cu_2_(OOC_6_H_4_Br)(OCH_3_)(bipy)_2_(ClO_4_)_2_] (**3**, Figure 1), using H_2_O_2_ (30% aq. solution) in water, under open air and at 70 °C. The di-nuclear complex proved to be an active catalyst for producing the corresponding aldehydes or ketones. Thus, benzyl alcohol and 1–phenylethanol were quantitatively oxidized in 6 h (entries 16 and 18, respectively, Table 1), with concomitant high turnover number (TON) values [12].

Complex **3** was generally found to be more effective as a catalyst for benzylic and cyclic alcohols than for 1-heptanol as a primary aliphatic alcohol (compare entries 16–19 of Table 1). Moreover, the selectivity of this catalytic system was tested between a primary and a secondary alcohol, or between a cyclic alcohol and an aliphatic alcohol. For the oxidation of the mixture of benzyl alcohol and 1–phenylethanol, 16% of benzaldehyde was obtained, together with 62% of acetophenone. Hence, this catalytic system shows a preference to act on secondary alcohols. The oxidation of a mixture of a linear secondary alcohol (2-butanol) and a cyclic (cyclo-pentanol) one resulted in lower conversions for both, compared to yields of 100% and 71% for 2-butanone and cyclopentanone obtained from experiments on a unique substrate. Thus, the experiments indicated that the competition between alcohols results in lower product yields compared to the pure substrates under the same conditions.

If an organic solvent must be used in an oxidation reaction, acetonitrile will be the chosen one by almost any researcher. In fact, acetonitrile is a medium-polarity solvent, miscible with water and a variety of organic solvents, able to dissolve a wide range of ionic, as well as nonpolar, compounds. More importantly, acetonitrile is usually inert under the oxidation conditions, even for the most drastic ones.

**Scheme 2:**
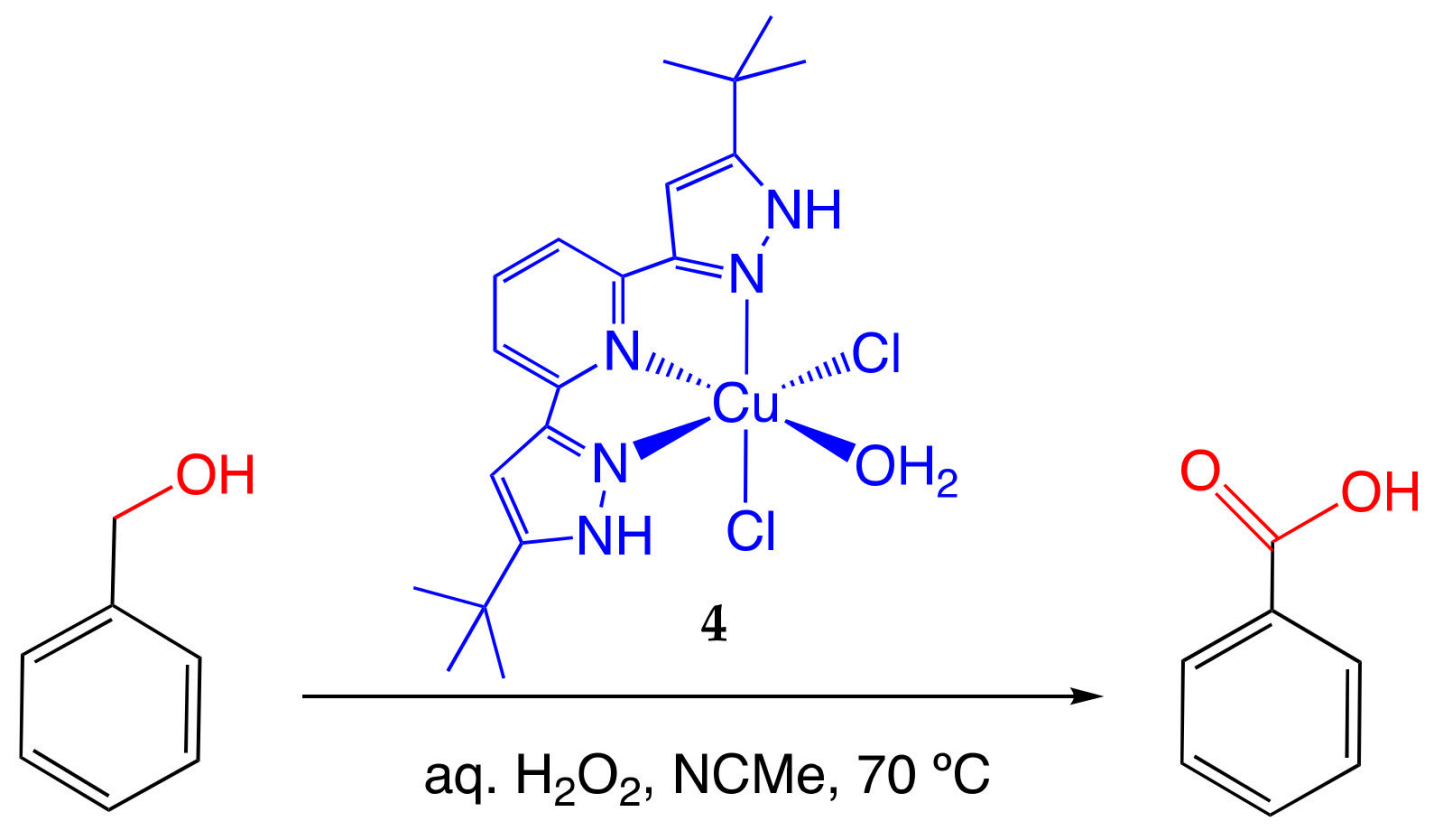
Direct oxidation of benzyl alcohol to benzoic acid catalyzed by the mononuclear Cu(II) complex [CuCl_2_(H_2_O)L] (**4**).

Thus, the catalytic potential of the recent mononuclear Cu(II) complex [CuCl_2_(H_2_O)L] (**4**, L = 2,6-bis(5-*tert*-butyl-1H-pyrazol-3-yl)pyridine) for the oxidation of benzyl alcohol was examined by Hu et al. [13]. The oxidation reactions were performed with H_2_O_2_ (30% aq. solution) in acetonitrile, at 70 °C. Interestingly, and in contrast with the majority of copper catalysts that afford mainly benzaldehyde under mild oxidation conditions, in the presence of **4**, after 1 h under the above mild peroxidative conditions, 98% of benzyl alcohol was converted to benzoic acid (96% yield, TON = 341, Scheme 2).

### 2.2. Oxidation with Tert-Butyl Hydroperoxide, Using Conventional Heating

Despite H_2_O_2_ being the peroxide of election in terms of green chemical practices, its decomposition often hampers its use at moderate/high temperatures. Thus, *tert-*butyl hydroperoxide (TBHP), commercially available in a 70% aqueous solution, appears usually as a suitable (although producing butanol) and economic alternative.

In 2017, Shul′pin and coworkers [14] applied the hexanuclear Cu(II)-based phenylsilsesquioxane complex [(PhSiO_1.5_)_10_(CuO)_6_(HO_0.5_)_2_(C_12_H_8_N_2_)_2_] (**5**) as catalyst for the oxidation of 1-phenylethanol with *tert*-butyl hydroperoxide (TBHP, 70% aq. solution) in acetonitrile, at 50 °C and in presence of HNO_3_, leading to 94% yield of acetophenone (TON = 600) after 14 h. Moreover, the cage-like compound [(PhSiO_1,5_)_6_]_2_[CuO]_4_[NaO_0.5_]_4_(dppmO_2_)_2_ (**6**), obtained very recently [15] by self-assembly synthesis, has also shown to be a very good catalyst for the oxidation of alcohols in acetonitrile solution: 1-phenylethanol was oxidized by TBHP to acetophenone in an almost quantitative yield. In addition, complexes bearing cyclic germsesquioxanes [PhGeO_1,5_]_5_ and phenanthrolines, namely [(PhGeO_1.5_)_10_(CuO)_6_(HO_0.5_)_2_(C_12_H_8_N_2_)_2_](H_2_O)_2_ (**7**), exhibited high activity as pre-catalysts in homogeneous oxidations of cyclohexanol, 2-heptanol or 1-phenylethanol to the corresponding ketones, leading to yields up to 94% [16].

**Figure 2:**
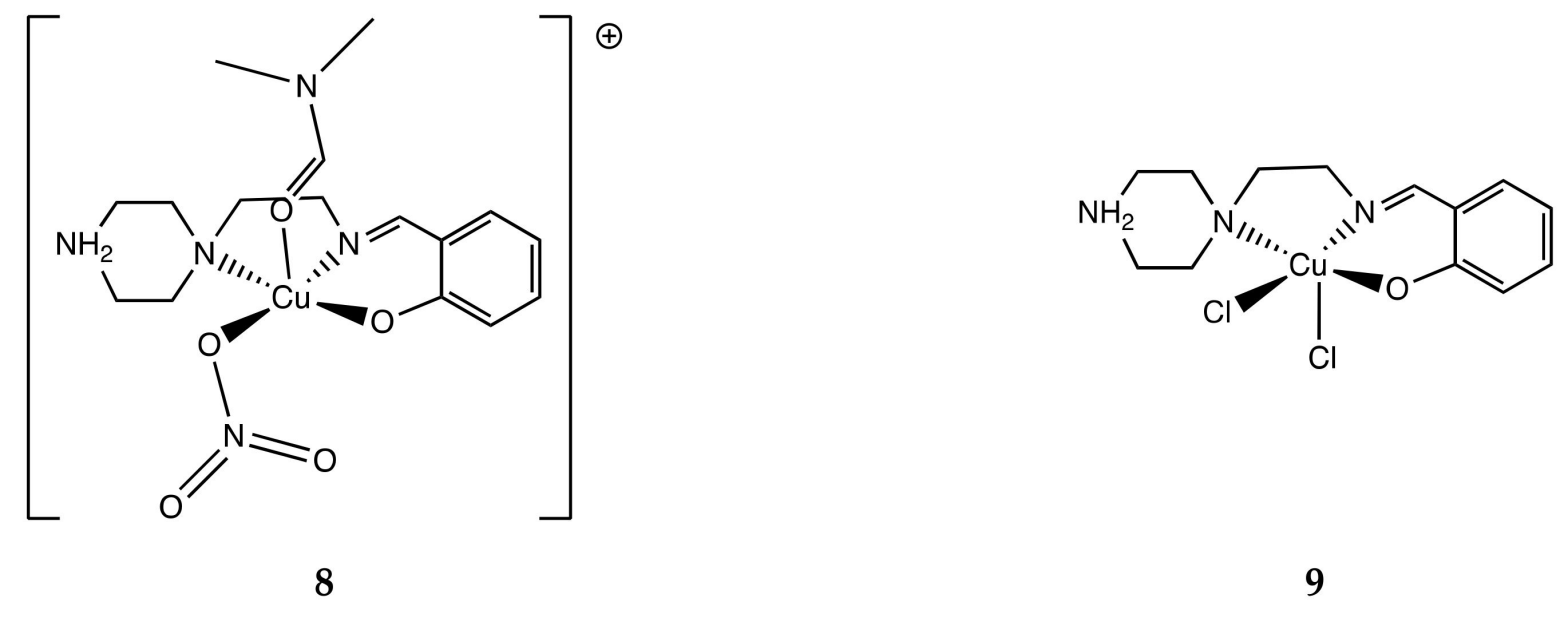
Mononuclear Cu(II) complexes [Cu(κ*ONN*’-HL)(NO_3_)(DMF)](NO_3_)∙H_2_O (**8**) and [Cu(κ*ONN*’-HL)Cl_2_]∙½DMSO (**9**).

The mononuclear copper(II) complexes [Cu(κ*ONN*’-HL)(NO_3_)(DMF)][NO_3_]∙H_2_O (**8**, DMF = dimethylformamide) and [Cu(κ*ONN*’-HL)Cl_2_]∙½DMSO (**9**) (Figure 2) obtained from the Schiff base aminoalcohol HL (product of condensation of salicylic aldehyde and aminoethylpiperazine), were reported by Nesterov et al. [17] and tested as potential catalysts (0.05–0.1 mol% vs. substrate) for the oxidation of 1-phenylethanol to acetophenone with TBHP (2 eq. vs. substrate, 70% aq. solution). The reaction performed under additive-free conditions led to rather low (12% or 23% for **8** or **9**, respectively) yields of acetophenone and TON values (246–468). The addition of either TEMPO free radical or *N*-hydroxyphthalimide (NHPI, precursor of phthalimido-*N*-oxyl radical) increased the yield and TON values for **8**, whereas no significant influence of these additives was detected for **9**. Whereas the presence of HNO_3_ hampered the oxidation reaction, the addition of K_2_CO_3_ significantly increased its rate. Moreover, the reaction strongly depended on the temperature: The activity of both **8** and **9** increased with the temperature, being the highest yield (62% of acetophenone, for **8**) and TON (620, for **8**) values reached at 100 °C. Thus, in spite of the efforts of the authors, **8** and **9** remained as moderately active catalysts for the oxidation of 1-phenyl ethanol.

### 2.3. Microwave Assisted Oxidation with Tert-Butyl Hydroperoxide

Another strategy followed by the researchers toward the improvement of the sustainability of the catalytic processes is the use of alternative energy sources where heating is required. Microwave (MW) irradiation turns out to be one of the most chosen alternative heating modes. In fact, MW irradiation promotes faster and more efficient internal heating through direct interaction between microwave energy and the reaction mixture components (solvents, reagents or catalysts). Therefore, while traditional conduction heating causes the mixture in contact with the reaction vessel wall to be heated first, microwave-radiation heating raises the temperature of the entire reaction system simultaneously. Compared to conventional heating, the use of microwave radiation has been shown to lead to a drastic reduction in reaction times, an increase in yield and selectivity observed in a number of chemical reactions, while also allowing a reduction or even elimination, in some cases, of the use of hazardous solvents.

**Figure 3:**
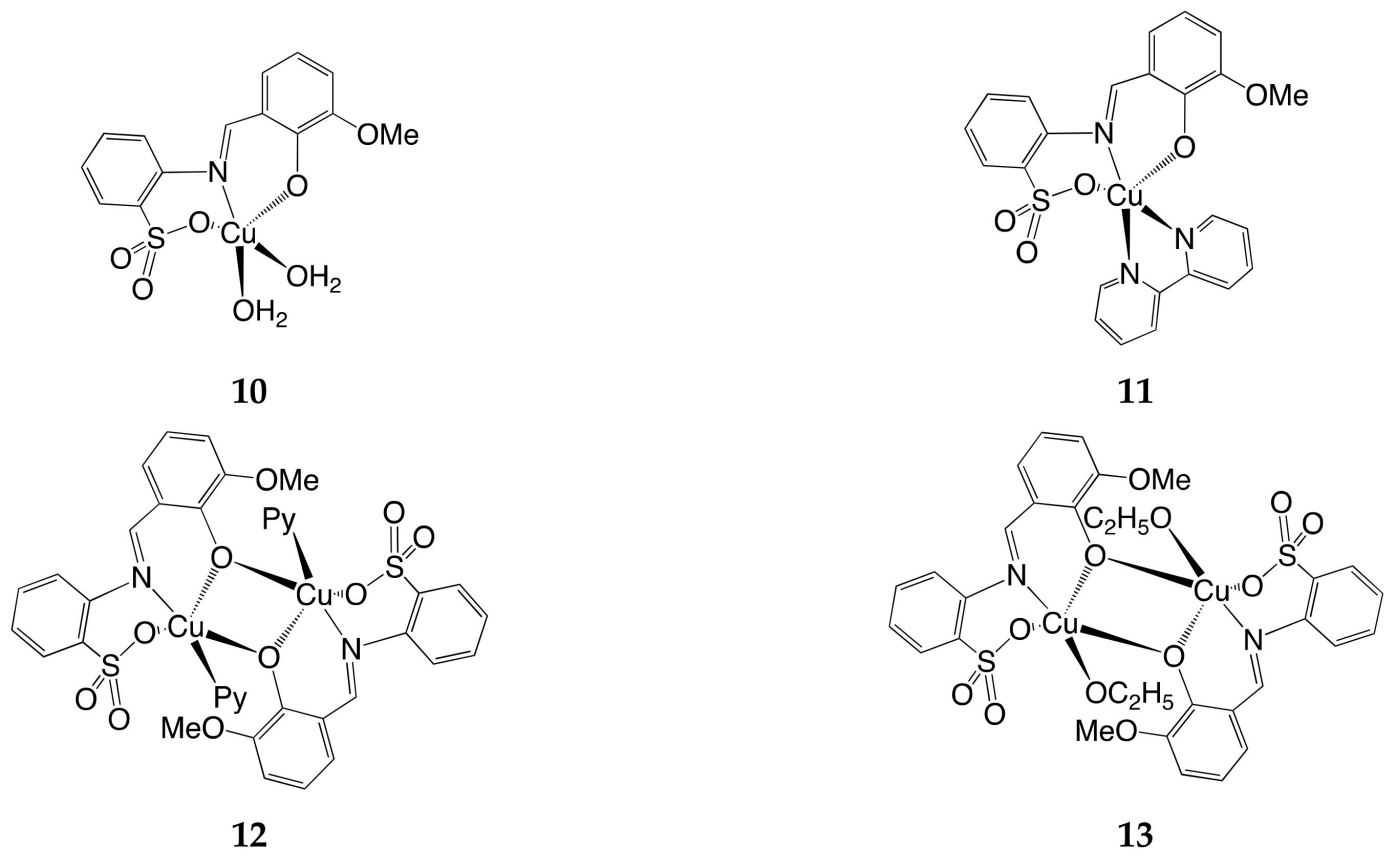
Mononuclear Cu(II) complexes [CuL(H_2_O)_2_] (**10**) and [CuL(bipy)]·DMF·H_2_O (**11**), and the diphenoxo-bridged dicopper compounds [CuL(py)]_2_ (**12**) and [CuL(EtOH)]_2_·2H_2_O (**13**).

In 2015, Martins et al. reported [18] the use of the mononuclear complexes [CuL(H_2_O)_2_] (**10**, H_2_L = 2-[(2-hydroxy-3-methoxyphenyl)methylideneamino]benzenesulfonic acid) and [CuL(bipy)]·DMF·H_2_O (**11**), as well as of the diphenoxo-bridged dicopper compounds [CuL(py)]_2_ (**12**, py = pyridine) and [CuL(EtOH)]_2_·2H_2_O (**13**) (Figure 3), as selective catalysts for the neat-microwave-assisted oxidation of primary and secondary alcohols, under additive-free conditions. Moreover, 1-Phenylethanol was quantitatively converted into acetophenone (entry 5, Table 2), in the presence of **12** with turnover frequency (TOF) values up to 126.7 min^−1^, after 20 min of MW irradiation. Moreover, the almost quantitative conversion of benzyl alcohol to benzaldehyde (99% yield, TOF 16.5 min^−1^, entry 6, Table 2) was also considered noticeable by the authors, since previous methods employing Cu(II) catalysts and TBHP under MW irradiation led to lower benzaldehyde yields (up to 75%) and also needed higher reaction temperatures (up to 150 °C) [16]. The selectivity of **13** to oxidize the alcohol relative to the ene function was evaluated by using cinnamyl alcohol as substrate and water as solvent, under the optimized conditions. Remarkably, cinnamaldehyde (85% yield, TOF 14.2 min^−1^) was the only product detected, clearly revealing the preference of **13** to oxidize the alcohol function.

**Figure 4:**
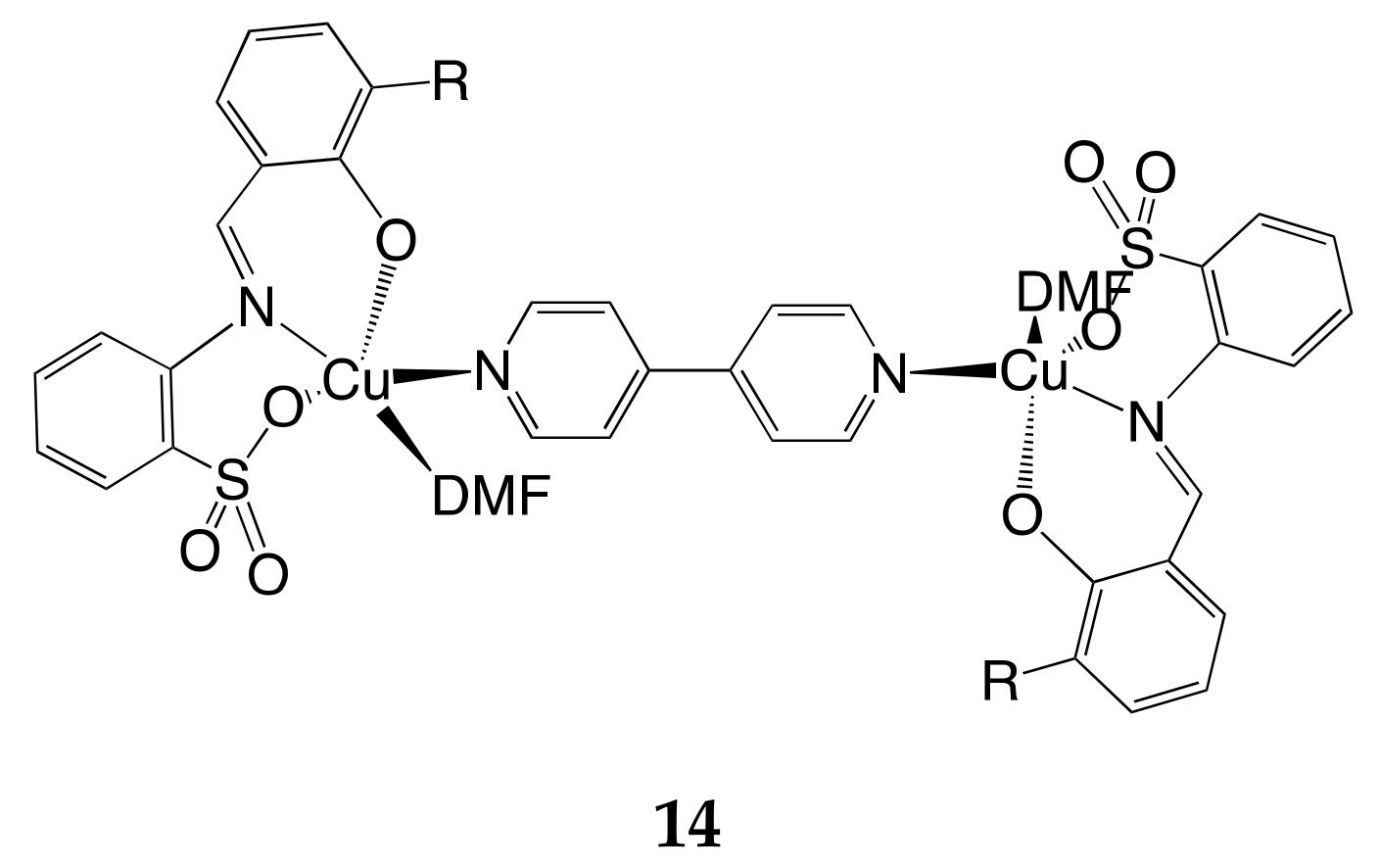

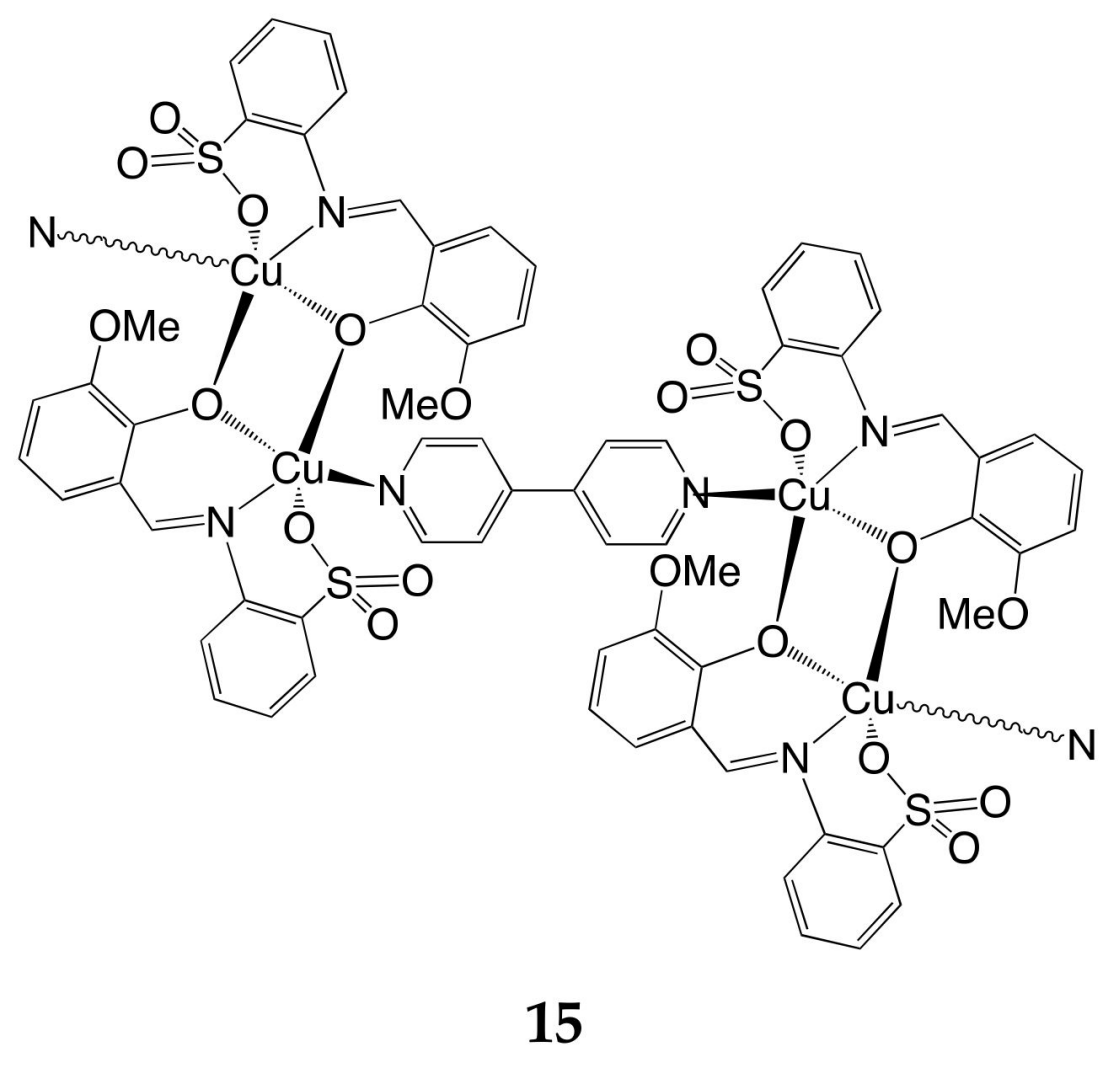
Dicopper(II) complex [Cu_2_(L-κ*ONO*^´^)_2_(µ-4,4′-bipy)(DMF)_2_] (**14**) and the dicopper(II)-based coordination polymer [Cu_2_(µ-L-1κ*ONO*´:2κO)_2_(µ-4,4′-bipy)]_n_·nH_2_O·nDMF (**15**).

An extension of this study to the preparation of further copper complexes with the above types of ligands was reported by the same authors in 2017 [19] (Figure 4). The dicopper(II) complex [Cu^2^(L-κ*ONO*^’^)_2_(µ-4,4′-bipy)(DMF)_2_] (**14**, H_2_L = 2-[(2-hydroxy-3-methoxyphenyl)methylideneamino] benzenesulfonic acid) and the coordination polymer [Cu_2_(µ-L-1κ*ONO*′:2κO)_2_(µ-4,4′-bipy)]_n_·nH_2_O·nDMF (**15**) were tested as catalysts for the oxidation of primary and secondary alcohols. A good catalytic activity for the neat and additive-free MW-assisted oxidation of the aliphatic cyclohexanol (up to 90% yield of cyclohexanone) after 30 min of low power MW irradiation (entries 9 and 12, Table 2) was achieved by these dinuclear Cu(II) compounds. Under the same reaction conditions, the conversion of the aryl 1-phenylethanol or benzyl alcohol occurred in to a much less extent (up to 58% and 49% for acetophenone and benzaldehyde, respectively, for **15**, entries 13 and 14, Table 2).

**Figure 5:**
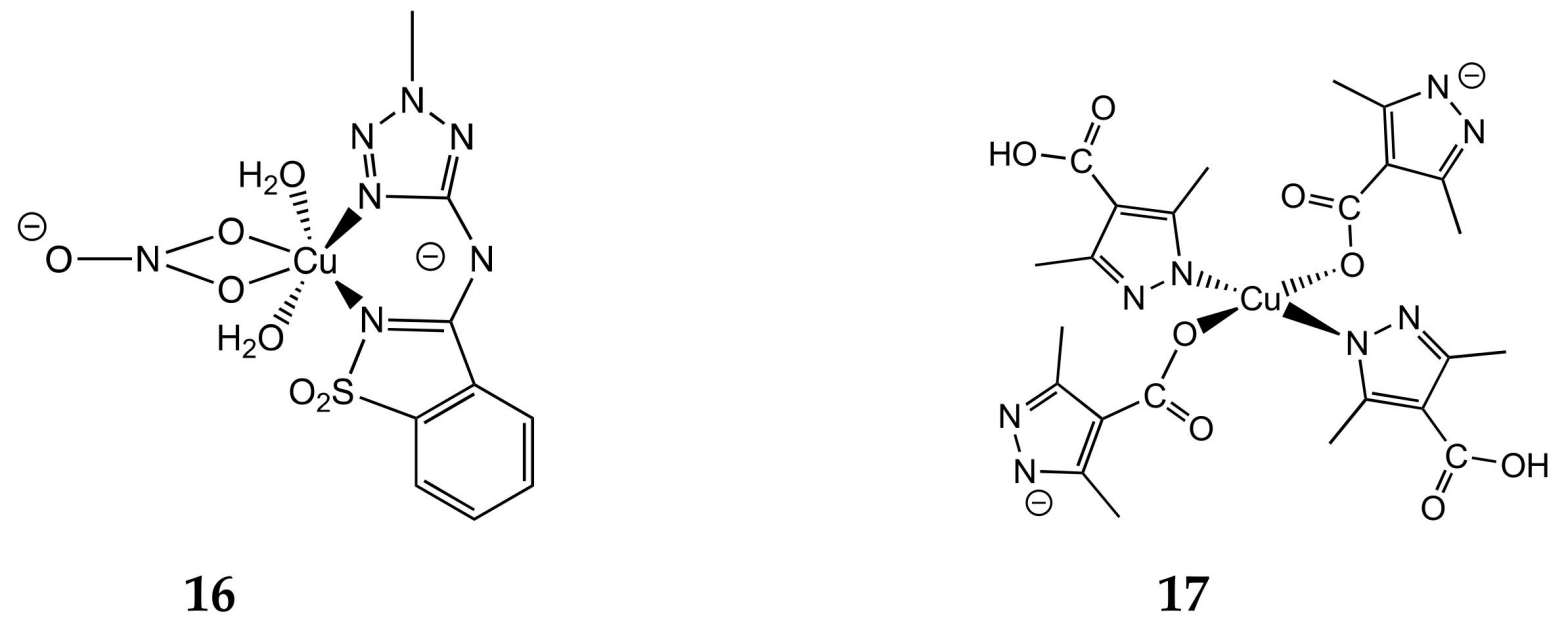
Mononuclear Cu(II) complexes (**16**) and [Cu(Hdmpzc)_2_] (**17**).

Frija et al. developed [20] a new catalytic system for the MW-assisted neat oxidation of alcohols into the corresponding carbonyl derivatives. Thus, a mononuclear Cu(II) complex comprising the 2-methyltetrazole-saccharinate bidentate *N,N*-chelating ligand (**16**, Figure 5) was synthesized and found to exhibit a broad functional group compatibility, allowing efficient and selective conversion of a variety of secondary benzylic, allylic and aliphatic alcohols, including those with the functional groups susceptible to oxidation, such as internal alkenes or primary/secondary amines. Generally, the reactions were complete after 10–15 min of MW irradiation, at 80–130 °C, with high TON and TOF values (up to 91.7 and 183.3 min^−1^, respectively) in additive-free and co-oxidant-free conditions. No significant conversion differences were observed for benzylic, allylic or aliphatic substrates. Moreover, the authors also found TEMPO acting as an inhibitor for **16** under the above peroxidative conditions.

In 2016, a different type of alcohol oxidation catalyst was reported by Galli and coworkers [21]: the thermal stable copper azolate/carboxylate compound [Cu(Hdmpzc)_2_] (**17**, H_2_dmpzc = 3,5–dimethyl-1H-pyrazol-4-carboxylic acid, Figure 5). Complex **17** was found to act as a remarkably active catalyst for the microwave-assisted oxidation of neat 1-phenylethanol to acetophenone as the only product. In fact, yields up to 99% at 120 °C were achieved in only 0.5 h of MW irradiation (entry 19, Table 2). The hypothesis of recovering this homogeneous catalyst at the end of the first reaction run was hampered by its decomposition under the oxidation conditions.

**Figure 6:**
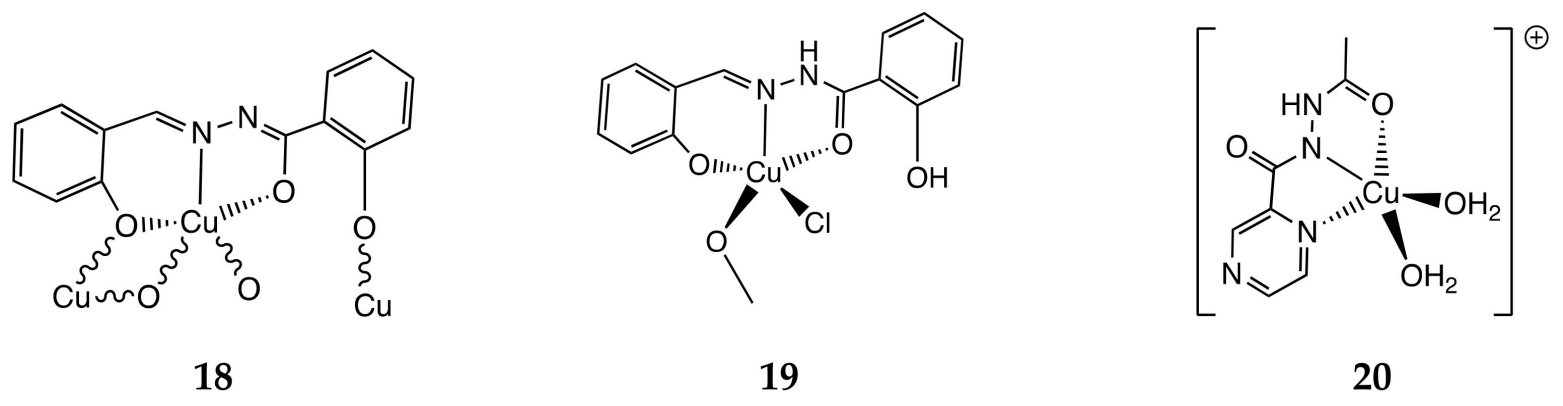
Cu(II) complexes [Cu(1κ*NOO*’,2κ*O*’,3κ*O*′′-L)]_n_ (**18**), [Cu(κ*NOO*′-HL)Cl(CH_3_OH)] (**19**) and [Cu((k*NN*′*O*-HL)(H_2_O)_2_](NO_3_) (**20**).

Cu(II) complexes in two different tautomeric forms (*keto* and *enol*) derived from the aroylhydrazone Schiff base 2-hydroxy(2-hydroxybenzylidene)benzohydrazide (H_2_L) were reported by Sutradhar et al. [22] (Figure 6). While the compound with the *enol* form of the ligand exists as the 1D polymer [Cu(1κ*NOO*’,2κ*O*’,3κ*O*”-L)]_n_ (**18**), the one bearing the *keto* tautomer ligand occurs as the monomer [Cu(κ*NOO*’-HL)Cl(CH_3_OH)] (**19**). Both complexes act as selective catalysts for the microwave-assisted oxidation of 1-phenylethanol with TBHP. Moreover, **19** exhibits the highest activity leading selectively to a maximum acetophenone yield of 92% (entry 22, Table 2).

Very recently, the same authors reported [22] the catalytic activity of the related new mononuclear Cu(II) complex [Cu((k*NN*′*O*-HL)(H_2_O)_2_](NO_3_) (**20**, H_2_L = *N*-acetylpyrazine-2-carbohydrazide, Figure 6) for the oxidation of primary and secondary aryl alcohols (benzyl alcohol and 1-phenylethanol), and the secondary aliphatic cyclohexanol, using TBHP under added solvent-free microwave irradiation conditions. Compound **20** led to a maximum acetophenone yield of 91% (entry 22, Table 2) in the presence of TEMPO as promoter for the oxidation of 1-phenylethanol, whereas 68% of cyclohexanone was attained in the same conditions. This behavior favoring the oxidation of aryl alcohols relative to the aliphatic ones, contrasts with that of the previously disclosed for complexes **14** and **15** (see above).

**Figure 7:**
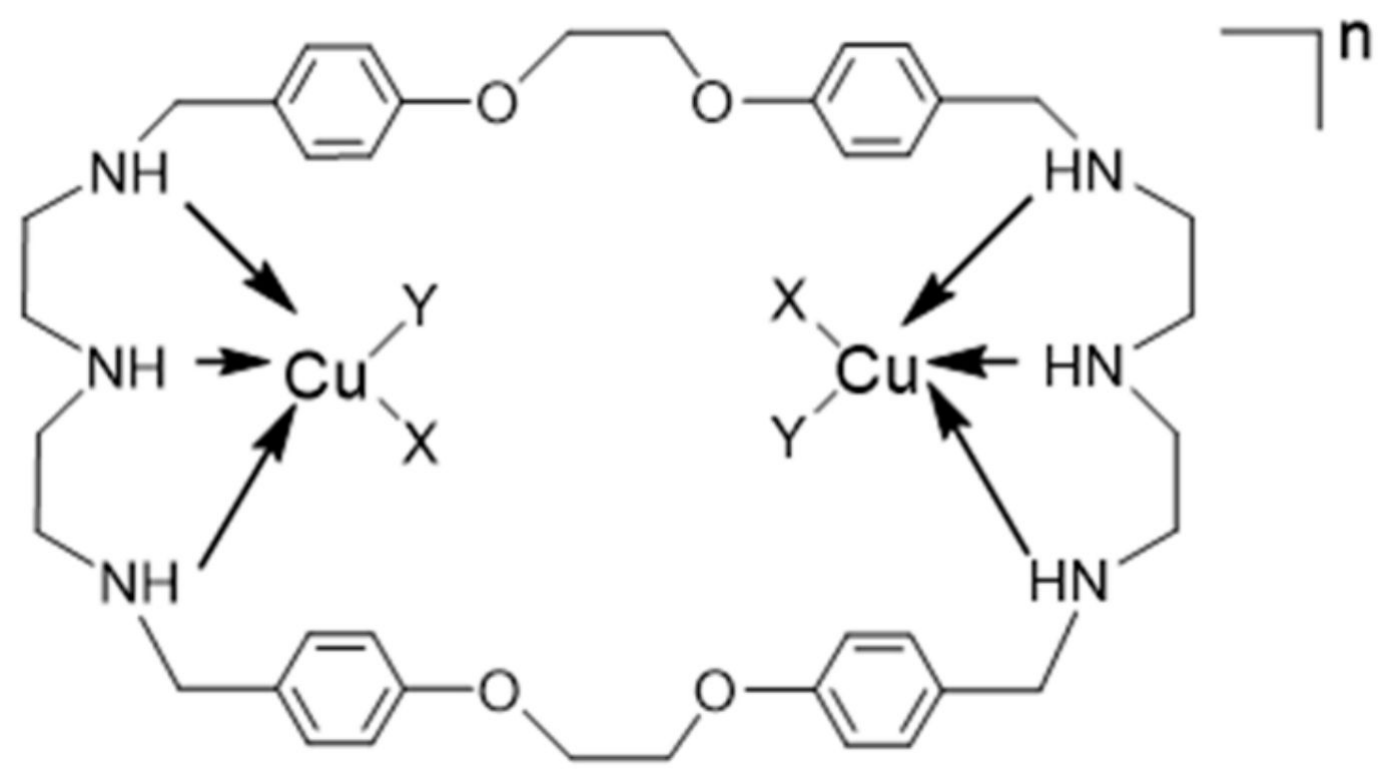
Dicopper(II) macrocyclic compounds [n = 2+, X = DMF, Y = OSO_2_CF_3_ (**21**); X = DMF, Y = OSO_2_C_6_H_4_Me (**22**); X = DMF, Y = ONO_2_ (**23**); X = DMF, Y = OClO_3_ (**24**); X = H_2_O, Y = OCOPh (**25**); n = 0, X = Y = OCOMe (**26**)].

Recently, Ma et al. reported [24] new dinuclear compounds [Cu_2_(OSO_2_CF_3_)_2_(DMF)_2_L] [SO_3_CF_3_]_2_ (**21**, L = 1,4,19,22,25,40-hexaaza-10,13,31,34-tetraoxa6,14,27,35(1,4)-tetrabenzenacyclopentacontane), [Cu_2_(*p*-OSO_2_C_6_H_4_Me)_2_L(DMF)_2_](SO_3_C_6_H_4_Me)_2_ (**22**), [Cu_2_(ONO_2_)_2_L(DMF)_2_](NO_3_)_2_ (**23**), [Cu_2_(OClO_3_)_2_(DMF)_2_L](ClO_4_)_2_ (**24**), [Cu_2_(OOCPh)_2_L(H_2_O)_2_](O_2_CPh)_2_ (**25**) and [Cu_2_(OOCMe)_4_L] (**26**) (Figure 7), able to catalyze the microwave-assisted oxidation of neat 1-phenylethanol by TBHP, leading to acetophenone yields up to 99% and TOF values of 18.3 min^−1^, after 0.5 h, without any additive. Complexes **22** and **26** present the highest catalytic activity (entries 27 and 31, Table 2) among these with the polyaza N_6_O_4_ macrocyclic ligand.

Interestingly, such complexes (**21**–**26**) also act as catalysts for the aerobic oxidation of primary alcohols in the presence of TEMPO in alkaline solution (see Section 3, below).

**Scheme 3:**
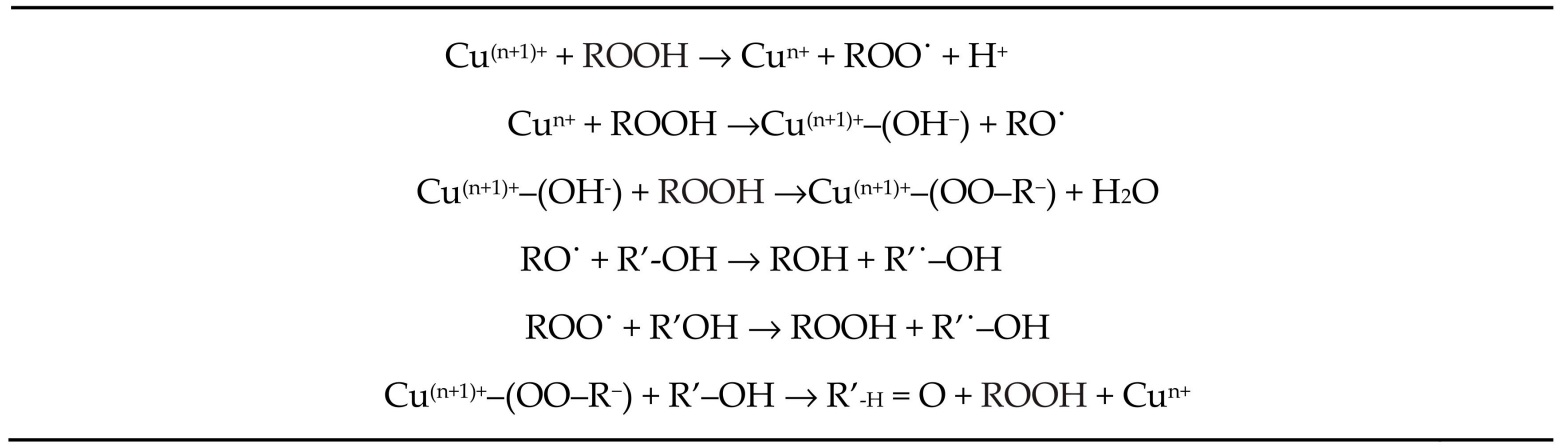
Possible pathways for the Cu-assisted catalytic peroxidative oxidation of an alcohol (R’-OH).

To the best of our knowledge, during the 2015–2020 period, no significant advances regarding the mechanism of Cu-catalyzed oxidation of alcohols with peroxide reagents were reported. The current well-established radical mechanism (Scheme 3) proposes the generation of ROO^●^ and RO^●^ radicals upon Cu-assisted oxidation and reduction of the peroxide (ROOH), which will behave as hydrogen abstractors from the alcohol to proceed the oxidation reaction.

## 3. Aerobic Oxidation of Alcohols

The use of molecular oxygen as oxidant, in combination with a metal catalyst, has practical advantages due to the favorable economics associated with O_2_ and the formation of environmentally benign by-products: water and hydrogen peroxide. Copper would seem to be an appropriate choice for the selective catalytic oxidation of alcohols with dioxygen since it comprises the catalytic center in a variety of enzymes, e.g., galactose oxidase (which catalyzes this conversion in vivo) [25]. Thus, a process that may address green chemistry matters (see Section 1) would be the implementation of a Cu-catalyst in combination with molecular oxygen as the oxidant. The low costs, ready availability of copper and the interesting spectroscopic properties of its coordination compounds resulted in the development of numerous Cu–based catalysts for the aerobic oxidation of alcohols.

All the recently reported aerobic Cu-based catalytic systems for alcohols oxidation, manly primary alcohols, encompass the 2,6,6-tetramethylpiperidine-1-oxyl radical (TEMPO). The nitroxyl radical, by itself, cannot catalyze the oxidation of alcohols by dioxygen, relying on the assistance of a co-catalyst to activate the oxidant [5]. The most used ones are first-row transition-metal coordination compounds where copper complexes bearing different N-donor ligands account for the prime ones. In fact, as follows, the combination of a Cu-complex with TEMPO and dioxygen leads to very active catalytic systems for the selective oxidation of primary alcohols, in particular of benzyl alcohol to benzaldehyde.

The di-nuclear Cu-compounds **21–26**, recently disclosed by Ma et al. [24], besides catalyzing the MW-assisted peroxidative oxidation of 1-phenylethanol (see Section 2.3), in combination with TEMPO and in alkaline aq. solution, exhibit efficient catalytic activity for the aerobic oxidation of different alcohols to the corresponding aldehydes (yields up to 99% and TON values up 232 for benzaldehyde, entries 1–6, Table 3) after 20 h at 70 °C. The formation of the corresponding carboxylic acid was not observed conceivably on account of the propensity of TEMPO for scavenging free radicals, acting as an effective radical trap, avoiding the auto-oxidation of aldehydes. Moreover, the reaction strongly depended on the temperature and for temperatures higher than 70 °C, aldehyde yield values decrease conceivably due to the lower solubility of dioxygen in the reaction solution.

**Figure 8:**
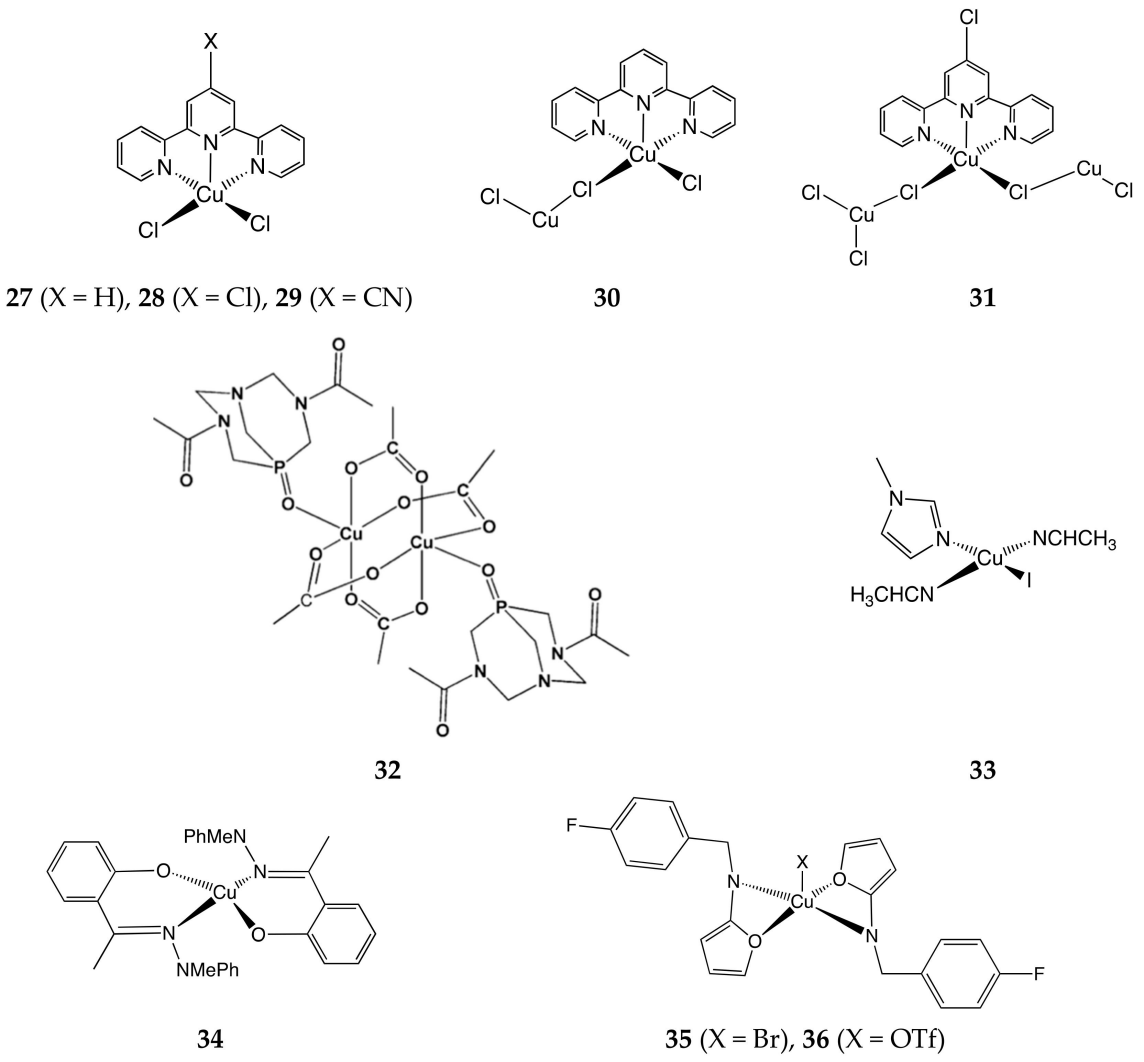
Copper complexes [CuCl_2_(4′-Xtpy)] [X = H (**27**), Cl (**28**) or CN (**29**)], [Cu^I^Cu^II^(tpy)Cl_3_] (**30**), [Cu(4′-Cltpy)Cl(CuCl_2_)]*_n_* (**31**), [Cu^II^(µ-CH_3_COO)_2_(*k*O-DAPTA = O)]_2_ (**32**)**,** [CuI(NMI)(NCMe)_2_] (**33**), [Cu(L^NNMePh^)_2_] (**34**) and [CuX(C_11_H_8_FNO)_2_] [ X = Br (**35**) or X = OTf (**36**)].

The catalytic activity of Cu(II) complexes bearing 2,2:6,2-terpyridine derivatives [Cu(4′-Xtpy)Cl_2_] [tpy = terpyridine; X = H (**27**), Cl (**28**) or CN (**29**), Figure 8] was evaluated by Zhang et al. [26] for the oxidation of benzylic alcohol to benzaldehyde by air, in water and in the presence of TEMPO and K_2_CO_3_. Moreover, **27** led to 53% yield of benzaldehyde after 20 h at 70 °C. In contrast, **28** catalyzed the reaction under the same conditions with a higher yield (74%), whereas the conversion achieved with **29** was much lower (37%) than that for the related catalysts **27** and **28**. Interestingly, **27**–**29** acted as efficient catalysts (benzaldehyde yields 90%–99%, entries 7–9, Table 3) when, to the above reactions, 4-dimethylaminopyridine (DMAP) was added as an extra base. Moreover, the authors assessed the influence of TEMPO radical on the formation of new reactive intermediates during the catalytic oxidation reactions by introducing it into the reaction medium of the complex’s formation. These coordination reactions in the presence of TEMPO led to the new mixed-valence Cu^I^Cu^II^ supramolecular assembles [Cu^I^Cu^II^(tpy)Cl_3_] (**30**) and [Cu(4-Cltpy)Cl(CuCl_2_)]*_n_* (**31**). Complexes **30** and **31** exhibited higher catalytic activity than those of the mononuclear complexes under milder conditions: Quantitative conversions (TOF ca. 0.3 min^−1^) were obtained within 5 h in both cases (entries 10 and 11, Table 3), even though the reactions were performed at room temperature.

**Figure 9:**
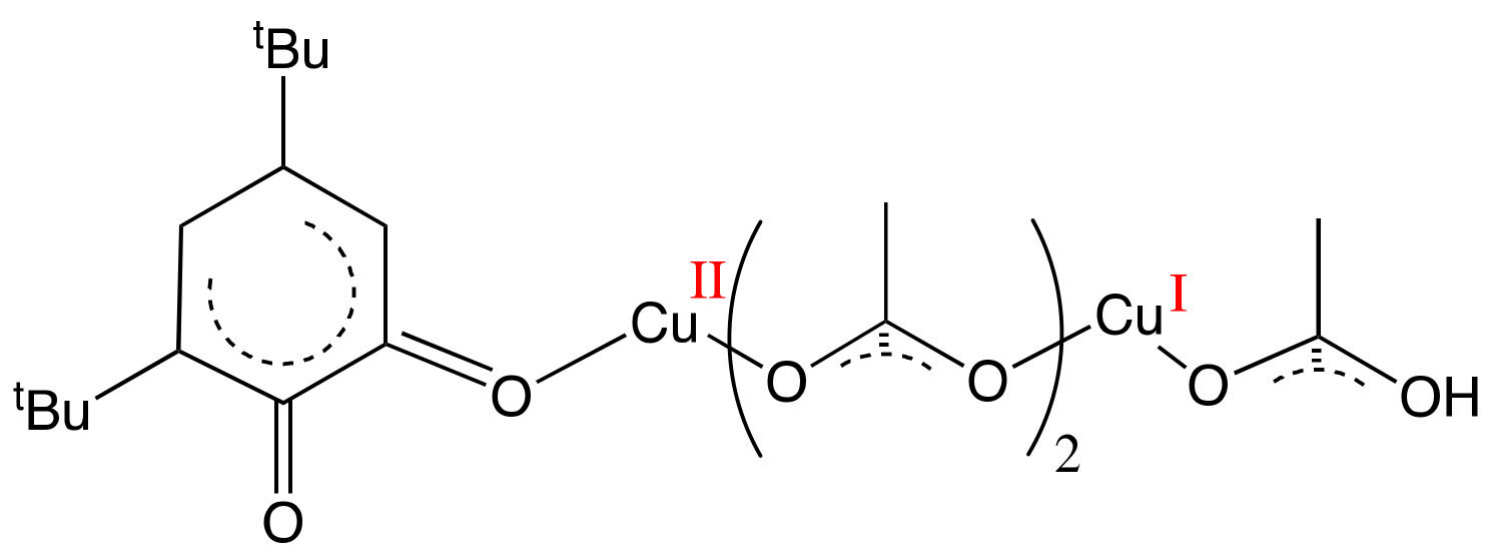
Proposed intermediate formed in the oxidation of 3,5-di-tert-butylcatechol catalyzed by complex **32** (the DAPTA = O ligands are omitted for clarity).

Pombeiro and coworkers reported [27] the first example of a copper complex based on 3,7-diacetyl-1,3,7-triaza-5-phosphabicyclo [3.3.1]nonane-5-oxide (DAPTA = O) ligand, [Cu(µ-CH_3_COO)_2_(*k*O-DAPTA = O)]_2_ (**32**, Figure 8). The catalytic activity of **32** was evaluated for the aerobic TEMPO-mediated oxidation of benzyl alcohol (entries 12–14, Table 3), and of 3,5-di-tert-butylcatechol (model substrate for catechol oxidase) [28]. The kinetic data fit the Michaelis–Menten equation, and the rate constant obtained is among the highest obtained for 3,5-di-tert-butylcatechol (using di-nuclear copper(II) complexes). DFT calculations suggested a mixed valence Cu^II^/Cu^I^ complex intermediate, (Figure 9) in which the spin electron density is mostly concentrated at one of the Cu atoms and at the DAPTA = O ligand.

The knowledge that external bases have an effect on the catalytic activity, as well as on the selectivity and stability of the catalytic systems, has led to the development of new ones able to operate in the absence of an added base [29]. Theoretical and experimental studies revealed that appropriate ligands enhance the electron density of the Cu center (e.g., by ligand-to-metal charge transfer (LMCT)), leading to a complex with a suitable alkalinity and suggesting that a basic anion in the Cu-complex could be more efficient for promoting the oxidation reaction than an external base added.

Liu et al. reported [30] the homogeneous Cu(I)/NMI/TEMPO catalytic system (NMI = methyl imidazole) for the aerobic oxidation of 1-octanol (99% of octanal after 24 h) and other alcohols (e.g., benzyl alcohol, entry 15, Table 3) into the corresponding aldehydes, at room temperature, in acetonitrile. The catalytic species was found to be the Cu(I) complex [CuI(NMI)(NCMe)_2_] (**33**, Figure 8), where the labile solvent binding to the copper center is crucial to ensure both oxygen and alcohol coordination. Moreover, NMI, as ligand, improved the Cu(I) center electron density and endorsed O_2_ coordination. In particular, the system exhibited strong oxidizing ability to quantitative conversion of benzylic alcohols (entry 15, Table 3), regardless of the substituents on the phenyl ring, and of allylic alcohols into aldehydes.

The catalytic activity of the copper(II) complex [Cu(L^NNMePh^)_2_] (**34**) [L^NNMePh^ = κ^2^-*N*,*O*-2-(1-(2-methyl-2-phenylhydrazono)ethyl)phenolate, Figure 8] in combination with TEMPO was evaluated, by Conejo et al. [31], for the oxidation of benzyl alcohol by air, in toluene. Very low yields of benzaldehyde were attained (up to 14% after 6 h at 60 °C, or 10% after 18 h at r.t., entries 16 and 17, respectively, Table 3).

Repo and coworkers disclosed [32] a new Cu(I) complex bearing *N*-(4–fluorophenyl)-1-(furan-2-yl)methanimine (C_11_H_8_FNO) [CuBr(C_11_H_8_FNO)_2_] (**35**, Figure 8) acting as catalyst for the selective oxidation of several primary alcohols to aldehydes under ambient (r.t.), conditions (with air, TEMPO and NMI in acetonitrile). **35** exhibited high activity, leading to, e.g., nearly quantitative yields of aldehydes (e.g., 1 h of oxidation of benzyl alcohol at r.t. yielded 99% of benzaldehyde, entry 18, Table 3). Moreover, replacing the coordinated bromide by triflate, to generate [Cu(OTf)(C_11_H_8_FNO)_2_] (**36**) (OTf = CF_3_SO_3_, Figure 8), led to an increase in the product yields of the primary alcohols oxidation. This was considered by the authors to be a remarkable catalytic result, as the oxidation reactions occurred in open air (rather than under pure oxygen) and at room temperature. Secondary alcohols, such as 2-octanol, were hardly oxidized, due to the steric bulkiness of TEMPO.

In situ formed Cu(I)/TEMPO-based catalytic systems have also been reported for the successful oxidation of several alcohol substrates, including those previously found as problematic.

Swarts et al. [33] reported a in situ Cu(I)/TEMPO/NMI catalyst system formed with a bis(pyridyl)-*N*-alkylamine. Quantitative conversion of 1-octanol was achieved, even though the reaction rate for this ligand was slightly slower than for the previously used bipy. A variety of primary aliphatic, allylic, benzylic and heterocyclic alcohols was also converted, with excellent yields of the corresponding aldehydes, whereas secondary alcohols could not be oxidized on account of the steric effect exerted during the (bulky) TEMPO-mediated hydrogen abstraction from the alcohol.

Recently, a highly efficient Cu(I)/TEMPO catalytic system for the (fast) aerobic oxidation of an aliphatic alcohol (intermediate in a pharmaceutical synthesis) to the corresponding aldehyde was developed by Roberts et al. [34], which appears suitable for safe large-scale application in a batch reactor. A number of key by-products (some not previously reported for Cu(I)/nitroxyl-catalyzed aerobic alcohol oxidation) that affect catalyst performance and aldehyde yield were identified with the help of kinetic information and allowed the scale up of the oxidation reaction, with bubbling air, in a batch reactor.

The mechanistic pathway of aerobic oxidations of alcohols catalyzed by Cu/TEMPO systems has been investigated by several research groups [35,36,37,38,39,40], and these investigations have led to partially contradicting mechanistic proposals involving either TEMPO+, TEMPO· or TEMPO-H as the reactive intermediate; moreover, this was recently thoroughly revised by Swarts et al. [28].

## 4. Conclusions

Important advances are being made in the design strategies for selective catalytic oxidations of primary and secondary alcohols. Clean oxidants, such as dioxygen or peroxides in aqueous, mono- or bi-phasic systems, as well as neat oxidations, are significantly increasing. In addition, taking advantage of non-conventional reaction conditions, such as microwave-assisted oxidations, or selecting catalysts of inexpensive and environmentally benign first-row-transition metals, with copper as one of the most preferred, are noticeable trends. Thus, Cu/peroxide- or Cu/O_2_-based catalytic systems could constitute attractive alternatives to traditional oxidation methods, although challenges to find the optimal catalyst for oxidizing both primary and secondary alcohols remain. Nevertheless, considering the pace of advances in recent years, it is expectable that such issues will be soon addressed.

## Figures and Tables

**Table 1:** Catalytic performance of copper complexes **1**–**3** in the oxidation of alcohols by hydrogen peroxide in water.

Entry	Catalyst	Time (h)	Substrate	Product	Yield (%)	Ref.
1	**1** *^a^*	6	benzyl alcohol	benzaldehyde	97	
2		6	cyclohexanol	cyclohexanone	28	
3		24	cyclohexanol	cyclohexanone	52	
4		6	1-phenylethanol	acetophenone	73	
5		24	1-phenylethanol	acetophenone	100	[10]
6		6	1-heptanol	1-heptanone	12	
7		24	2-octanol	2-octanone	11	
8		6	3-pentanol	3-pentanone	29	
9		24	3-pentanol	3-pentanone	100	
10		6	benzyl alcohol	benzaldehyde	71	
11		6	cyclohexanol	cyclohexanone	29	
12	**2** *^b^*	24	cyclohexanol	cyclohexanone	100	[11]
13		6	1-phenylethanol	acetophenone	86	
14		24	1-phenylethanol	acetophenone	100	
15		24	2-octanol	2-octanone	11	
16	**3** *^c^*		benzyl alcohol	benzaldehyde	100	
17			cyclohexanol	cyclohexanone	53	
18		6	1-phenylethanol	acetophenone	100	
19			1-heptanol	1-heptanone	17	
20			cyclo-pentanol	cyclopentanone	71	[12]

*^a^* Alcohol (0.76 mmol), catalyst ((0.1 mol% vs. alcohol), H_2_O_2_ (19.5 mmol). *^b^* Alcohol (0.93 mmol), catalyst (0.1 mol% vs. alcohol), H_2_O_2_ (0.2 mmol). *^c^* Alcohol (0.57 mmol), catalyst (0.1 mol% vs. alcohol), H_2_O_2_ (19.5 mmol).

**Table 2:** Catalytic performances for copper complexes **10**–**26** in the neat MW-assisted oxidation of primary and secondary alcohols, using TBHP as the oxidant.

Entry	Catalyst	Time (min.)	Substrate	Product	T (°C)	Yield (%)	Ref.
1	**10** *^a^*	20	1-phenylethanol	acetophenone	100	64	[18]
2		150	benzyl alcohol	benzaldehyde		58	
3	**11** *^a^*	20	1-phenylethanol	acetophenone		52	
4		150	benzyl alcohol	benzaldehyde		79	
5	**12** *^a^*	20	1-phenylethanol	acetophenone		100	
6		150	benzyl alcohol	benzaldehyde		99	
7	**13** *^a^*	20	1-phenylethanol	acetophenone		94	
8		150	benzyl alcohol	benzaldehyde		92	
9	**14** *^b^*	30	cyclohexanol	cyclohexanone	60	85	[19]
10			1-phenylethanol	acetophenone		55	
11			benzyl alcohol	benzaldehyde		41	
12	**15** *^b^*		cyclohexanol	cyclohexanone		90	
13			1-phenylethanol	acetophenone		58	
14			benzyl alcohol	benzaldehyde		49	
15	**16** *^c^*	5	1-phenylethanol	acetophenone	120	100	[20]
16		6			130	95	
17		2			130	72	
18		10			120	60	
19	**17** *^d^*	30			120	99	[21]
20	**18** *^e^*	60			120	81	[22]
21	**19** *^e^*					92	
22 *^f^*	**20**	60	1-phenylethanol	acetophenone	120	91	[23]
23 *^g^*			benzyl alcohol	benzaldehyde		41	
24 *^f^*			cyclohexanol	cyclohexanone		68	
25	**21** *^h^*	30	1-phenylethanol	acetophenone	120	79	[24]
26	**22** *^h^*					66	
27						99	
28	**23** *^h^*					81	
29	**24** *^h^*					64	
30	**25** *^h^*					68	
31	**26** *^h^*					99	

*^a^* Alcohol (2.5 mmol), catalyst (0.04 mol% vs. alcohol), TBHP (2 eq., 70% in H_2_O), 10 W MW irradiation. *^b^* Alcohol (2.5 mmol), catalyst (0.2 mol% vs. alcohol), TBHP (2 eq., 70% in H_2_O), 10 W MW irradiation. *^c^* Alcohol (2.5 mmol), catalyst (0.2 mol% vs. alcohol), TBHP (2 eq., 70% in H_2_O), 20–50 W MW irradiation. *^d^* Alcohol (2.5 mmol), catalyst (0.4 mol% vs. alcohol), TBHP (2 eq., 70% in H_2_O), 20 W MW irradiation, n(TEMPO)/n(catalyst) = 3.*^e^*Alcohol (2.5 mmol), catalyst (0.4 mol% vs. alcohol), TBHP (2 eq., 70% in H_2_O), 5–15 W MW irradiation.*^f^* Alcohol (5 mmol), catalyst (0.2 mol% vs. alcohol), TBHP (2 eq., 70% in H_2_O), 5 W MW irradiation, n(TEMPO)/n(catalyst) = 25. *^g^* Alcohol (5 mmol), catalyst (0.2 mol% vs. alcohol), TBHP (2 eq., 70% in H_2_O), 5 W MW irradiation, n(Ph_2_NH)/n(catalyst) = 25. *^h^* Alcohol (5 mmol), catalyst (0.2 mol% vs. alcohol), TBHP (2 eq., 70% in H_2_O), 5–15 W MW irradiation.

**Table 3:** Catalytic performances of copper complexes **21**–**35** for the selective aerobic oxidation of benzyl alcohol to benzaldehyde.

Entry	Catalyst	Time (h)	T (°C)	Yield (%)	Ref.
1	**21**	20 *^a^*	70	97	[24]
2	**22**			99	
3	**23**			94	
4	**24**			79	
5	**25**			84	
6	**26**			99	
7	**27**	20 *^b^*	70	95	[26]
8	**28**			>99	
9	**29**			90	
10	**30**	5 *^b^*	25	>99	
11	**31**			>99	
12	**32** *^c^*	6	80	41	[27]
13		21		78	
14		48		89	
15	**33** *^d^*	24	25	99	[29]
16	**34** *^e^*	18	25	10	[31]
17		6	60	14	
18	**35** *^f^*	1	25	>99	[32]

*^a^* Alcohol (1.5 mmol), catalyst (0.5 mol% vs. alcohol), TEMPO (5 mol% vs. alcohol), K_2_CO_3_ (0.1 M). *^b^* Alcohol (1.0 mmol), catalyst (0.01 mmol), DMAP (0.050 mmol), TEMPO (0.050 mmol), K_2_CO_3_ (0.2 M), water. *^c^* Alcohol (0.5 mmol), catalyst (5 mol% vs. alcohol), TEMPO (5 mol%), K_2_CO_3_ (0.1 M). *^d^* Alcohol (5 mmol), catalyst (0.25 mmol), TEMPO (5 mol%), acetonitrile. *^e^* Alcohol (1.0 mmol), catalyst (5 mol% vs. alcohol), TEMPO (5 mol%), NMI (10 mol%), acetonitrile. *^f^* Alcohol (1.0 mmol), catalyst (5 mol% vs. alcohol), TEMPO (5 mol%), NMI (10 mol%), acetonitrile, open air.
